# Optofluidic multiplex detection of single SARS-CoV-2 and influenza A antigens using a novel bright fluorescent probe assay

**DOI:** 10.1073/pnas.2103480118

**Published:** 2021-05-04

**Authors:** Alexandra Stambaugh, Joshua W. Parks, Matthew A. Stott, Gopikrishnan G. Meena, Aaron R. Hawkins, Holger Schmidt

**Affiliations:** ^a^School of Engineering, University of California, Santa Cruz, CA 95064;; ^b^Electrical and Computer Engineering Department, Brigham Young University, Provo, UT 84602

**Keywords:** optofluidics, single antigen detection, biosensing, integrated optics

## Abstract

The urgency for the development of a sensitive, specific, and rapid point-of-care diagnostic test has deepened during the ongoing COVID-19 pandemic. Here, we introduce an ultrasensitive chip-based antigen test with single protein biomarker sensitivity for the differentiated detection of both severe acute respiratory syndrome coronavirus 2 (SARS-CoV-2) and influenza A antigens in nasopharyngeal swab samples at diagnostically relevant concentrations. The single-antigen assay is enabled by synthesizing a brightly fluorescent reporter probe, which is incorporated into a bead-based solid-phase extraction assay centered on an antibody sandwich protocol for the capture of target antigens. After optimization of the probe release for detection using ultraviolet light, the full assay is validated with both SARS-CoV-2 and influenza A antigens from clinical nasopharyngeal swab samples (PCR-negative spiked with target antigens). Spectrally multiplexed detection of both targets is implemented by multispot excitation on a multimode interference waveguide platform, and detection at 30 ng/mL with single-antigen sensitivity is reported.

## Significance

This work introduces an ultrasensitive single protein capture and detection technique based on a bright fluorescent reporter probe that is sensed on a photonic chip with integrated microfluidics. We perform differentiated detection of single SARS-CoV-2 and influenza A antigens at clinically relevant concentrations from clinical nasal swab materials. This ultrasensitive capture and detection technique could one day be realized as a tool for molecular diagnostics at the point of care.

The extensive impact of the current coronavirus disease (COVID-19) pandemic caused by the SARS-CoV-2 (severe acute respiratory syndrome coronavirus 2) virus has shone a spotlight on the dire need for diagnostic testing on a heretofore unseen scale to help detect and contain the spread of infectious diseases (1). This virus has already infected over 100 million people and caused millions of deaths. With diagnosed cases reaching over 700,000 globally per day, these numbers are only going to increase (2). Ideally, such a test would be accurate, sensitive, quick, and low complexity so that it could be deployable as close to the point of care as possible. Development of a diagnostic test that simultaneously fulfills all these desirable criteria has been a longstanding challenge, which has been recognized with other viral outbreaks such as the 2009 influenza A (H1N1) pdm09 pandemic (3–5) as well as with chronic diseases such as hepatitis C that are widespread in underdeveloped regions (6, 7). Molecular diagnostics approaches that target disease biomarkers on the molecular level have emerged as the clear choice to detect both viral and bacterial infections. The gold standard is reverse transcriptase quantitative PCR (RT-qPCR), which amplifies small amounts of the target’s genomic material and provides a real-time, highly sensitive, and specific readout for analysis (8, 9). During the current pandemic, for example, patient samples are analyzed by PCR in centralized laboratories, which adds logistical challenges and prolongs turnaround times. While PCR systems are increasingly being designed and deployed at the point of care (10, 11), rapid point-of care testing at scale is currently done with antigen tests that detect viral proteins. However, such tests suffer from insufficient sensitivity and are often considered inadequate for clinical decision-making absent a secondary confirmation with a more reliable technique (12–15). Therefore, an antigen test that overcomes this sensitivity limitation while retaining its advantages in speed and complexity is extremely desirable. Additionally, it may be desirable to detect different molecular target types (e.g., viral nucleic acids and host-produced antibodies) from the same sample and with a single platform, and neither of the approaches described above offers this flexibility. Finally, multiplex detection of different viral targets is an essential component of many diagnostic panels because different diseases can present with similar symptoms. For example, standard tests for influenza and other respiratory infections screen for four to eight pathogens simultaneously (16, 17).

Here, we report the development of an antigen test for multiplex detection with single-protein biomarker sensitivity and its validation with simultaneous diagnosis of both SARS-CoV-2 and influenza A antigens in PCR-negative clinical nasopharyngeal swab samples spiked with target antigens at clinically relevant concentrations. Our approach is based on an optofluidic lab-on-a-chip (LoC) device with single molecule sensitivity. LoC is considered to be a promising next-generation platform for medical diagnostics that enables instruments with compact footprints and miniaturized microfluidic sample handling that require only small sample volumes (18). Optofluidics enhances the levels of integration by incorporating photonic principles and has produced a number of promising devices (19, 20) that were used to detect single biological nanoparticles such as virions and bead-based capture complexes of many nucleic acid and protein antigen targets. These methods include resonance shifts upon target binding (21), direct fluorescence imaging with a smart phone camera (22), and interferometric imaging of targets on a functionalized surface (23). We have developed a biophotonic analysis platform based on Antiresonant Reflecting Optical Waveguides (ARROWs) that uses orthogonally intersecting liquid-core analyte-carrying ARROWs and excitation solid-core ARROWs to perform amplification-free fluorescence detection of single biomolecules in flow (24, 25). These silicon-based chips can be integrated with a programmable microfluidic sample preparation chip in order to create a single, chip-scale system for rapid sample-to-answer analysis (26–28). Target multiplexing was implemented by exciting different targets with spectrally and/or spatially varying multispot light patterns generated by multimode interference (MMI) waveguides (29). When these were intersected with single or multiple analyte waveguide channels, the target fluorescence signal reflects the different spatial excitation patterns and up to 7× multiplexing has been demonstrated (30–35). Critically, the flow-based detection on the waveguide chip is target agnostic and has enabled detection of a wide variety of biomolecules including liposomes, virions, nucleic acids, ribosomes, and proteins. However, single-protein detection had remained elusive up to now because, unlike nucleic acids and virions, it was challenging to label individual targets with a sufficient number of fluorophores for LoC detection.

Here, we develop a single-antigen assay on the ARROW platform by synthesizing a brightly fluorescent reporter probe that is compatible with a bead-based solid-phase extraction protocol and an antibody-based sandwich assay for specific detection of the target antigens. Optimization of the probe release for detection using ultraviolet (UV) light is discussed, and the full assay is validated with both SARS-CoV-2 and influenza A antigens. Finally, multiplex detection of both target types at 30 ng/mL from nasal swab samples with single-antigen sensitivity is reported, demonstrating the potential of this approach for use as an ultrasensitive test for protein biomarkers.

## Results

### Assay Design.

**Fig. 1.**
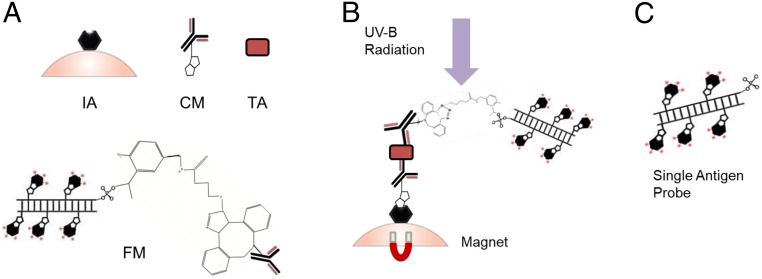
Single-antigen–probe assay design. (*A*) Components of the assay labeled: IA is the streptavidin-coated microsphere which is functionalized as the immobilization agent, CM is the biotinylated capture antibody which is the capture molecule, TA is the target antigen, and FM is the fluorescent reporter molecule which is the DCBO-labeled detection antibody bound to a 1-kB dsDNA probe with dye-labeled streptavidin reporters and a photo-cleavable azide molecule attached. (*B* and *C*) Full capture complex assembled as a sandwich assay on-bead (*B*); the PC linker on the probe is cleaved with UV-B radiation, and the probe is released, leaving a single probe per antigen for detection (*C*).

The single-antigen assay design is based on solid-phase target extraction used in conjunction with the ARROW platform for specific nucleic acid and protein antigen capture and detection. We break this assay into four components: an immobilization agent, capture molecule, target antigen, and fluorescence reporter. Here, the immobilization agent is a streptavidin-coated magnetic microsphere (Dynabeads MyOne Streptavidin T1, Thermo Fisher Scientific), which has quickly become the gold standard for the isolation and handling of biotinylated nucleic acids, antibodies, and other biotinylated ligands and targets. Microbead-based target extraction is both highly specific and target agnostic. The capture molecule in this assay is a biotinylated IgG antibody (anti-SARS-CoV-2 N protein for the SARS-CoV-2 capture assay and anti-influenza A antigen for the influenza A capture assay). The target antigen is a protein antigen from either SARS-CoV-2 or influenza A. Coronaviruses have four structural proteins: the Spike protein, the Envelope protein, the membrane protein, and the Nucleocapsid protein (36). For this assay, we use the Nucleocapsid protein as the target. The critical element for enabling single antigen sensitivity is a new fluorescence reporter molecule that is attached to a sandwich anti-SARS-CoV-2 N protein or anti-influenza A antigen IgG antibody. It consists of a Dibenzocyclooctyne (DBCO)-azide molecule bound to a bright fluorescent probe made from a 1-kilobase (kb) pair double-stranded DNA (dsDNA) backbone outfitted with biotinylated-deoxyuridine triphosphate (dUTP), which attaches dye-labeled monovalent streptavidin (mSA) molecules (see *Materials and Methods* section for details). Fig. 1*A* shows the individual components and how they connect to form the whole capture complex, which is shown in Fig. 1*B*.

The bright fluorescent probe molecule is made with a photo-cleavable spacer connected to an azide molecule, which gives the assay the functionality to be a single antigen detection technique. After the capture complex is formed, it is exposed to UV radiation that ranges from 300 to 350 nm (37). This breaks the photo-cleavable spacer and therefore separates the bright fluorescent probe from the capture complex bead that is pulled down with a magnet. Finally, the elute is collected postillumination for LoC analysis. This process is shown in Fig. 1*B*. The bright fluorescent probes (Fig. 1*C*) are then transferred to our ARROW optofluidic platform for single molecule detection. It is, therefore, ensured by design that every single reporter molecule detection event that is recorded from the ARROW optofluidic chip corresponds to a single-antigen–detection event, as one probe is equivalent to one antigen per the construction of the capture complex.

### UV Release Experiments.

Release experiments were first conducted in order to identify the optimal amount of time to irradiate the capture complex consisting of the bead-based target extraction sandwich assay with the bright fluorescent probe outfitted with the photo-cleavable spacer. The UV source is a Kernel KN-4003B UVB phototherapy lamp that emits far-field light at 311 nm. There is a trade-off in release experiments: irradiate the complex for too long an interval, and the UVB light (280 to 315 nm) will cause direct DNA damage to the dsDNA probe backbone; irradiate the complex for too short an interval, and the probes will be suboptimally released into solution. For 30, 45, 60, 75, and 90 s, 5 µL of capture complex was irradiated. An experiment was also done with longer irradiation times—up to 15 min—but there was damage done to the probe, and no signals were observed. The complex was pulled down postirradiation, and the supernatant was collected and diluted 1:10 in filtered 1× PBS buffer. A total of 5 µL of the diluted probe solution was transferred to the ARROW optofluidic platform for single molecule detection and analysis.

**Fig. 2.**
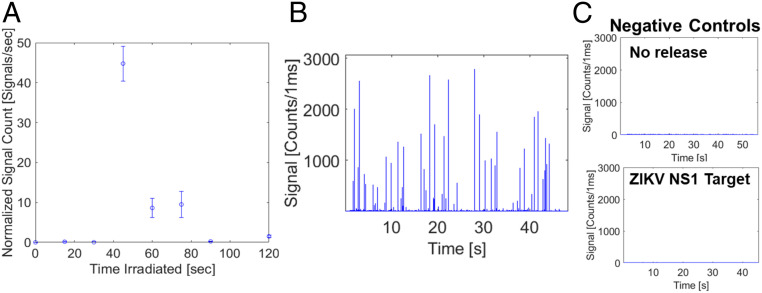
Results of probe release time experiments and controls. (*A*) Probe release time experiments measuring normalized fluorescence signals counted with varying release time for each probe. (*B*) Fluorescence particle trace of a SARS-CoV-2 N protein capture assay released for 45 s. (*C*) Fluorescence particle trace of a SARS-CoV-2 antigen capture assay released for 0 s (*Top*) and fluorescence particle trace of a mismatched target (ZIKV NS1 protein) in the SARS-CoV-2 antigen sandwich capture assay released for 45 s (*Bottom*).

**Fig. 3.**
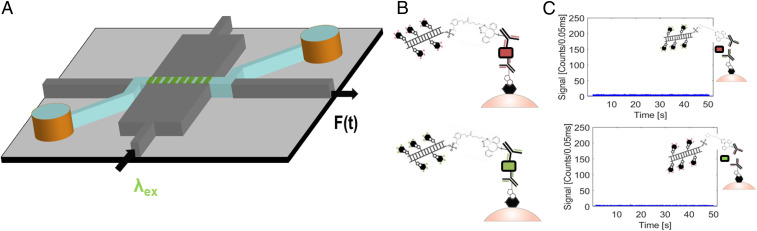
Setup for simultaneous dual detection of single SARS-CoV-2 and influenza A antigens. (*A*) Cartoon illustration of MMI-ARROW device with excited by λ1 = 556 nm (with excitation spot pattern shown, *n* = 8) and output fluorescence signal F(t). (*B*) Capture assays before release for Cy5-labeled SARS-CoV-2 N protein (*Top*) and Cy3-labeled influenza A antigen (*Bottom*). (*C*) Fluorescence particle trace of a mismatched target (SARS-CoV-2 N protein) in the influenza A antigen capture assay released for 45 s (*Top*) and fluorescence particle trace of a mismatched target (influenza A antigen) in the SARS-CoV-2 N protein capture assay released for 45 s (*Bottom*).

The postrelease probes were run through ARROW optofluidic chip outfitted with a single mode excitation waveguide and excited by a helium–neon (HeNe) laser at 633 nm (see Fig. 3*A* for the chip layout and *Materials and Methods* for a full description of the optical setup). Fig. 2*A* shows the results of this probe release time experiment with the normalized signal count (detected reporter molecules per time; see Fig. 2*B* plotted versus irradiation time. The average of at least three trials of this experiment is plotted in Fig. 2*A*, and the error bars represent the SE of the data for each irradiation time. There is a pronounced but highly reproducible peak around 45 s of irradiation time. This data point represents the average of five measurements and has an SD of 6%, ensuring good reproducibility of the assay for quantitative measurements. Fig. 2*B* shows the fluorescence particle trace for SARS-CoV-2 N protein antigen capture complex post 45 s release. It is evident that there are many fluorescence signals from individual probes flowing through the ARROW platform with a good average signal-to-noise ratio of nearly 80. This represents the direct detection of individual antigens on a chip and is the first principal result of this work. We note that the intensity of the signals varies based on the location of the probe within the cross section of the channel as previously observed (38). Fig. 2*C* shows the negative controls for the experiment, top with a 0 s irradiation time before pulldown and detection of the probes and bottom with a 45 s release time with a mismatched target, where the capture complex was made with Zika Virus Nonstructural 1 protein antigen, which is similar in size to the SARS-CoV-2 N protein antigen. Both negative controls show no fluorescence signals above the background, showing both excellent specificity in the assay and no errant fluorescence signals when the complex is not released.

### Multiplex Detection of SARS-CoV-2 N Protein and Influenza Antigen.

For most diagnostic assays, the ability to distinguish different pathogens that produce similar symptoms is highly desirable. In the case of COVID-19, differential diagnosis from influenza A is most important, particularly during the traditional flu season. Therefore, we implemented multiplex detection of SARS-CoV-2 and influenza A antigens on the ARROW chip by using an MMI excitation waveguide—a wide waveguide that supports numerous waveguide modes with different propagation constants and allows them to interfere with each other as they propagate down the length of the waveguide. At certain lengths at which the relative phases of these modes match up constructively, well-defined spot patterns are created (29). The optofluidic platform is designed such that the analyte-carrying liquid-core ARROW intersects the MMI waveguide where an integer numbers of spots are created. The dependence of the spot number (*N*) on excitation wavelength (*λ*) is derived from standard MMI theory and expressed by Eq. **1** (29):

$$N\;\left(\lambda \right)\;=\;\frac{n_{c}w^{2}}{\lambda L},$$ [1]

where *L* is the propagation length along the MMI waveguide (here: 1,975 µm), *w* is the width of the MMI waveguide (here: 75 µm), and *n*_*c*_ is the refractive index of the core of the MMI waveguide (here: 1.51). Fig. 3*A* shows a cartoon image of the optofluidic chip outfitted with the MMI waveguide. Note how the analyte ARROW fluidic channel intersects orthogonally with the excitation waveguide. Fluorescence signals are collected in-line with the ARROW liquid-core waveguide from a solid-core collection waveguide [F(t) in this; Fig. 3*A*] by downstream optics and are filtered by a penta-bandpass optical filter before being recorded by an avalanche photo diode. More details of the optical setup are shown in *SI Appendix*, Fig. S1. Fig. 3*B* shows the MMI spot patterns for excitation with λ_1_ = 556 nm and λ_2_ = 633 nm, with eight and seven well-defined spots, respectively.

Fig. 3*B* shows the completed capture constructs for the SARS-CoV-2 N protein antigen and the influenza A antigen. The SARS-CoV-2 N protein is captured onto a complex (top) that is labeled with an N-hydroxysuccinimide (NHS)-activated sulfo-Cyanine5 fluorophores (Cy5) probe and excited with 633 nm excitation light. The influenza A antigen is captured onto a complex (bottom) that is labeled with an NHS-activated sulfo-Cyanine3 fluorophores (Cy3) probe and excited with 556 nm excitation light. Fig. 3*C* demonstrates the specificity of the capture assay via two negative control experiments. The top figure shows a fluorescence particle trace of an influenza A capture complex made with the SARS-CoV-2 N protein antigen post–45-s UV release of the probe. The bottom figure shows the fluorescence particle trace of a SARS-CoV-2 N protein capture complex made with the influenza A antigen post–45-s UV release of the probe. In both fluorescence particle traces, there are no fluorescence signals above the background. This negative result was robust over multiple trial runs, which confirms the absence of false positive signals for this assay.

**Fig. 4.**
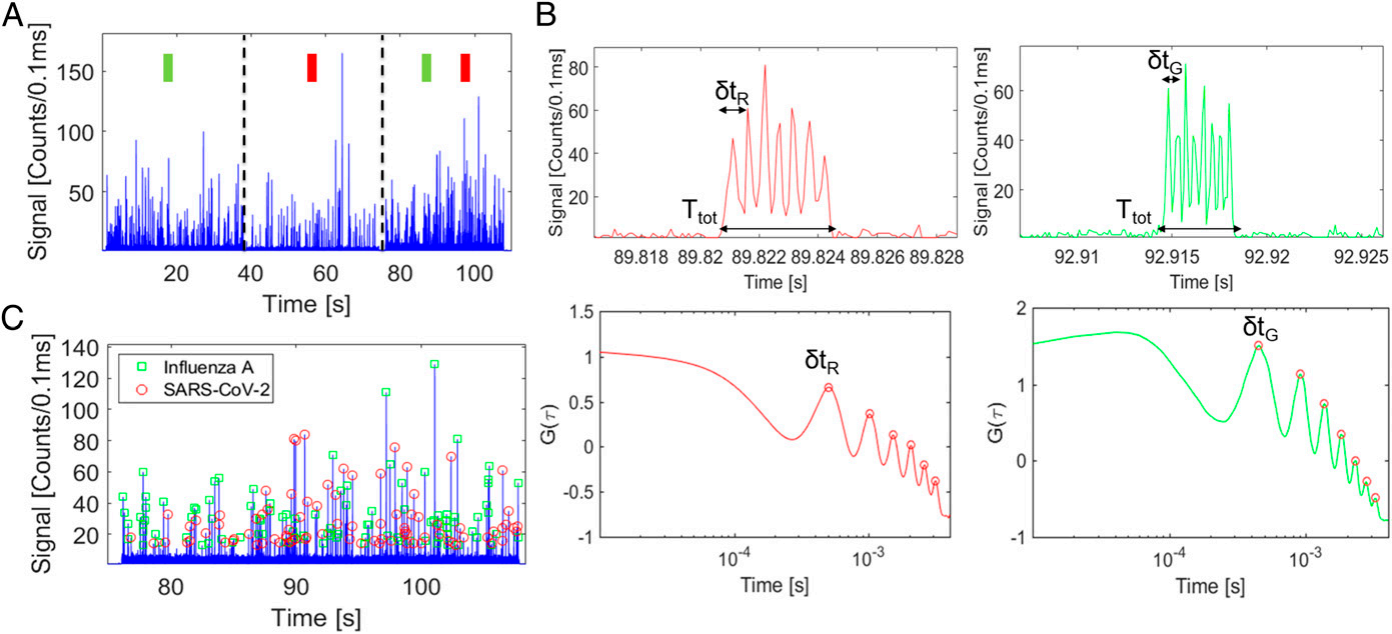
Results of simultaneous dual detection of single SARS-CoV-2 and influenza A antigens. (*A*) Fluorescence particle trace of both influenza A antigen and SARS-CoV-2 N protein single antigen capture probes post release, first excited with λ1= 556 nm only, then with λ2 = 633 nm, and then with both λ1 and λ2. (*B*) MMI spot patterns for both SARS-CoV-2 (*Top*) and influenza A (*Bottom*) probe signals, with autocorrelation shown and *δt* denoted for each signal. (*C*) Annotated multicolor fluorescence trace with influenza A (green squares) and SARS-CoV-2 (red circle) signals identified.

Finally, we turn to our core experiment—the simultaneous detection of both SARS-CoV-2 and influenza A antigens with single-target sensitivity from clinical (PCR-negative, deidentified) samples provided by the Molecular Diagnostics testing facility on the UC Santa Cruz campus. To this end, Fig. 4 details the multiplex detection experiment done with SARS-CoV-2–negative test swabs. Both the influenza A and SARS-CoV-2 N protein antigens were spiked into negative test swabs for SARS-CoV-2 to a clinically relevant concentration of 30 ng/mL, and the capture assay was performed. The capture complexes were subjected to 45 s of irradiation with UV light, and the probes in the elute were collected and diluted 1:10 in 1× PBS buffer. A total of 5 µL of that sample was pipetted into the inlet of the ARROW optofluidic chip for detection. Fig. 4*A* shows the fluorescence trace from this multiplex detection experiment. In the first ∼40 s of the trace, only the 556 nm excitation source was turned on, which only excited probes corresponding to single influenza A antigens. In the next 40 s, only the 633-nm excitation source was turned on, which only excited probes corresponding to single SARS-CoV-2 N protein antigens. In both cases, numerous signals originating from individual probes were detected with comparable rate and average intensity, confirming that the individual assays work and that both targets are indeed present. Fig. 4 *B*, *Top* shows close-ups of a SARS-CoV-2 signal with a seven-peak pattern created by the MMI excitation pattern at 633 nm and an influenza A signal with an eight-peak pattern created by the MMI excitation pattern at 556 nm. Fig. 4 *B*, *Bottom* shows the autocorrelation signals of those peaks, which exhibit *N*-1 maxima. The first maximum represents the time spacing *δt* between adjacent peaks in the time trace, which will be used for identification of the target. In the last ∼30 s of the trace in Fig. 4*A*, both excitation sources were turned on, fully demonstrating simultaneous multiplex detection of both probes corresponding to single influenza A and SARS-CoV-2 N protein antigens. Fig. 4*C* shows the fluorescence particle trace where both excitation sources are on in more detail. The trace is annotated such that all fluorescence signals are identified as either a SARS-CoV-2 N protein antigen peak or an influenza A antigen peak using multiple signal processing methods to differentiate the target specific fluorescence signals. To identify these events, we use a combination of two established methods. For the first, we extract the characteristic *δt* for each unidentified event from the first peak in the autocorrelation signal (Fig. 4 *B*, *Bottom*) and then perform a shift-multiply algorithm to classify it, described by Eq. 2 (39),

$$S\left(t,\delta t\right)=\;\overset{N-1}{\underset{m=1}{\prod }}F\left(t-m\cdot \delta t\right),$$ [2]

where *S*(*t*,*δt*) is the new shift-multiplied signal, *N* is the number of characteristic MMI spots, *F*(*t*) is the particle fluorescence signal, and *δt* is the characteristic time difference between each peak for an encoded signal extracted from the autocorrelation data and shown in Fig. 4*B*. *S*(*t*,*δt*) is enhanced when the particle trace is shifted by the correct peak count (*N*) and the correct time difference (*δt*). Because of background subtraction, it is quenched otherwise. Because we use a *δt* that is specific to each signal, we are able to obtain a velocity-independent confirmation of the signal. For the second method, we take the total time of each signal, *T*_*tot*_, and divide it by the characteristic *δt* of each signal. The result is a confirmation of *N*, the total number of peaks in the signal. This is described by Eq. **3** (31, 34):

$$N=\;\frac{T_{tot}}{\delta t}.$$ [3]

An event was declared identified as one of the two targets only if both methods yielded the same results. In the final multicolor trace, 53.5% of events detected were identified as influenza A antigens, and 46.5% of events were SARS-CoV-2 antigens. The rate of antigen detection for the 556-nm, single-color influenza A trace is 5.7 events per second, and the rate of antigen detection for the influenza A antigen in the multicolor trace is 3.1 events per second. Similarly, the rate of antigen detection for the 633-nm, single-color SARS-CoV-2 antigen is 2.6 events per second, while the rate of antigen detection for the SARS-CoV-2 antigen in the multicolor trace is 2.6 events per second. The rates of multicolor antigen detection are expected to be the same as the rates of single-color antigen detection on-chip, which we see empirically for the SARS-CoV-2 antigen detection and suggests that there is differentiated, single antigen detection in the multichromatic trace of the SARS-CoV-2 antigen. The discrepancies in the rates of the influenza A antigen detection between the single-color and multicolor traces is attributed to an increase in the threshold signal amplitude of the multicolor trace because of a higher background caused by both lasers being on simultaneously. For both single-color traces, the threshold was set to 5 photon counts per 0.1 ms, while the threshold for the multichromatic trace was set to 10 photon counts per 0.1 ms. The last piece of this puzzle lies in the distribution of peak amplitudes in *SI Appendix*, Fig. S2 *A* and *B*. The signal amplitude distribution for the single-color 556-nm portion of the trace skews to lower intensities than the distribution for the 633-nm trace, which suggests that there are more Cy3-labled influenza A antigen probe signals that are not counted because of the increase in threshold level. Additionally, the signal amplitude histograms for the probe release traces are similar in shape to the signal amplitude traces for single probes flown through the ARROW, which substantiates the claim that we are seeing single reporter detection. Finally, the assay was run in triplicate, yielding qualitatively identical results in the count rates with an SD of 4.6%, confirming the reproducibility of the full single-antigen detection assay.

## Discussion

We have designed and implemented a fluorescence assay for multiplex detection of viral antigens with single antigen sensitivity using direct counting of individual reporter particles on a compact optofluidic waveguide chip. This was enabled by the development of a bright fluorescent reporter probe that can be bound to and optically released from a secondary antibody. The reporter is compatible with a sandwich assay for specific extraction and labeling of targets on a microbead immobilization agent. The full assay was used to detect individual antigens from PCR-negative clinical swab samples, spiked with antigen targets at relevant concentrations, pointing the way toward a new generation of moderately complex, yet ultrasensitive, antigen tests. Both the bead-based extraction protocol and the flow-based, amplification-free detection of single particles are target agnostic and have been applied to amplification-free nucleic acid analysis. This introduces prospects for multitarget assays (e.g., proteins and nucleic acids) that may find use in infectious disease diagnostics, oncology, and clinical and fundamental research. In addition to novel and improved laboratory instruments, the chip-based optofluidic approach could also find use in point-of-care settings. This will require commercial product development that leverages the economies of scale of silicon microchip fabrication and photonic component suppliers. In addition, this process can be further advanced by chip-level integration of additional assay elements that have recently been demonstrated, including LoC sample processing (40) and light sources (41).

## Materials and Methods

### Experimental Setup.

The experimental setup implemented for multiplex detection of single antigens can be found in *SI Appendix*, Fig. S1. A 556-nm solid-state diode neodymium-doped yttrium aluminum garnet; Nd:Y_3_Al_5_O_12 _(SSD Nd:YAG) Laser (Shanghai Dream Laser Technology Co.) and a 633-nm HeNe laser (Melles Griot) are coupled into the same single-mode fiber. That single-mode fiber is butt-coupled into a single-mode excitation waveguide, which launches into the MMI waveguide, discussed above. The bright fluorescent probes traverse the MMI excitation volume and are excited by the spatially distributed MMI spot pattern and therefore generate multiple fluorescent peaks per the collected time domain signal. These signals are capture by the liquid-core ARROW, which is orthogonal to the MMI excitation waveguide (seen in Fig. 3*A*) and transmitted into the collection solid-core waveguide. Off-chip, the signal passes through an objective, is filtered by a penta-bandpass filter (FF01- 440/521/607/694/809–25, Semrock) to filter out the excitation wavelengths, and is finally recorded by a single photon avalanche diode (Excelitas Technologies). No additional downstream filters are required to spectrally separate the signals out because of the wavelength division multiplexing (WDM) created by the MMI waveguide.

### Bright Fluorescent Probe Synthesis.

The probe backbone is made from amplifying a 1-kB region of the pUC19 plasmid (New England Biolabs) using a 30-cycle PCR process. The forward primer is functionalized with a photo-cleavable spacer and azide molecule (IDT) to work as a linker in the capture assay. The PCR product is made in the presence of 10, 25, 50, and 75% biotinylated-dUTP in order to incorporate biotin into the 1-kB PCR probe backbone. The products were isolated using a PCR cleanup spin column (QIAgen QIAquick) and run through a 1% agarose gel, shown in *SI Appendix*, Fig. S3. Upon analysis of the amount of product made versus biotin incorporation, we settled into using 50% biotinylated-dUTP into our PCR product. mSA (Howarth Lab, Oxford University) was labeled using Cy3 and Cy5 according to manufacturer instructions (Lumiprobe). Briefly, the mSA samples were added to aliquots of dried Cyanine dyes and allowed to incubate at room temperature for 2 h. Samples were separated from free dye using Slide-a-lyzer 10 K molecular weight cutoff (MWCO) 0.1 mL dialysis units (Thermo Fisher Scientific) in 0.5 L dialysis buffer (1× PBS, Corning). Dialysis occurred overnight at 4 °C in the presence of gentle stirring. Aliquots were then transferred to Amicon Ultra Centrifugal Filter Units (Millipore Sigma) with a MWCO of 30 kDa and spin concentrated according to the manufacturer’s instructions to a final volume of 15 µL. The dye-labeled mSA was then added to aliquots of the probe backbone at a molar excess of ∼250, and the biotin-streptavidin reaction is then incubated at room temperature for up to 2 h. The excess dye-labeled streptavidin and probe-streptavidin complex are separated using a silica-gel membrane chromatography spin column (QIAquick PCR Purification Kit) which binds dsDNA and passes the unbound mSA through the column in the presence of high salt concentrations and elutes the dsDNA in low salt buffer. A 30-µL aliquot of ∼100-nM probe is then stored at 4 °C until further use in the assay.

### Functionalizing Capture and Detection Antibodies.

Capture antibodies are anti-SARS-CoV-2 N protein antibodies (HM1054, East Coast Bio) and anti-influenza A antigen (HM418, East Coast Bio) were ordered and labeled with biotin in house. An aliquot of antibody is added to a 20× molar excess of EZ-Link Sulfo-NHS-LC-LC-Biotin (Thermo Fisher Scientific) according to manufacturer’s instructions. The reaction is allowed to incubate at room temperature for up to 2 h. The conjugated antibody samples were separated from excess biotin using size exclusion chromatography via PD25 MiniTrap gel filtration columns (GE Healthcare) used according to the manufacture’s specifications. The samples eluted from the column were transferred to Amicon Ultra Centrifugal Filter Units (Millipore Sigma) with a MWCO of 100 kDa and spin concentrated according to the manufacturer’s instructions to a final volume of 30 µL. The concentrated antibody aliquots were then stored at 4 °C for further use. Detection-complex antibodies are anti-SARS-CoV-2 N protein antibodies (HM1055, East Coast Bio) and anti-influenza A antigen (HM419, East Coast Bio) were ordered and functionalized with DBCO in house. An aliquot of antibody is first dialyzed overnight to remove the sodium azide from antibody solution—because of the click-chemistry reaction between DBCO and azide—in a Slide-a-lyzer 10 K MWCO 0.1 mL dialysis unit (Thermo Fisher Scientific) in 0.5 L dialysis buffer (1× PBS, Corning). Dialysis occurred overnight at 4 °C in the presence of gentle stirring. Postdialysis, a 20× molar excess of DBCO-Sulfo-NHS ester (Millipore Sigma) is added to the dialyzed antibody and allowed to incubate at room temperature for up to 2 h. The conjugated antibody samples were separated from excess DBCO reagent using size exclusion chromatography via PD25 MiniTrap gel filtration columns (GE Healthcare) used according to the manufacture’s specifications. The samples eluted from the column were transferred to Amicon Ultra Centrifugal Filter Units (Millipore Sigma) with an MWCO of 100 kDa and spin concentrated according to the manufacturer’s instructions to a final volume of 30 µL. The concentrated antibody aliquots were then stored at 4 °C for further use.

### Full SARS-CoV-2 N Protein Assay Construction and Release.

An aliquot (0.05 mg) of Dynabeads MyOne T1 streptavidin-coated magnetic beads (Thermo Fisher Scientific) is washed three times in 1× PBS buffer and is incubated with 2 µg (∼5× molar excess) of biotinylated capture anti-SARS-CoV-2 N protein antibody (HM1054, East Coast Bio.) for 1 h at room temperature on a rotary mixer. Next, a magnet is used to separate the magnetic bead biotinylated antibody complex from the excess elute during a 4× washing step with 1× PBS. The solution is then resuspended in 5 µL 1× PBS. Added to the bead pulldown complex and incubated at 37° C for 2 h are 2 µg (∼5× molar excess) dibenzocyclooctyne (DBCO)-labeled anti-SARS-CoV-2 N protein antibodies (HM1055, East Coast Bio.) and up to 1 µg target (SARS-CoV-2 N protein model, East Coast Bio.) antigens. About 100-nM reporter probe molecules (1-kB pUC19 PCR product made with a photo-cleavable azide forward primer [IDT] and 50% biotinylated-dUTP and functionalized with Cy5-labeled mSA [Howarth Lab, Oxford University] so that there are ∼250 mSA/1-kB probe and up to 750 dye/probe) are then added to react with the DCBO-labeled antibody on the complex and allowed to incubate at room temperature for 1 h on a rotary mixer and then at 4 °C overnight. The capture assay is subject to two times wash step in 1× PBS to wash away excess unbound assay components and is then resuspended in 50 µL 1× PBS. An aliquot of 5 µL is then subject to UVB (311 nm) light for 45 s, then pulled down by a magnet, and 4 µL of the elute (probes) is added to 36 µL 1× PBS. A 5-µL aliquot of the sample is transferred to the ARROW platform for detection.

Influenza antigen capture assays are made similarly. The target is the influenza A antigen (East Coast Bio), the capture antibodies are anti-influenza A antigen antibodies (HM418, East Coast Bio), and the detection antibodies are anti-influenza A antigen antibodies (HM419, East Coast Bio).

Samples for the negative swab antigen tests were from nasal swabs were tested at the University of California, Santa Cruz (UCSC) Molecular Diagnostics Laboratory. Antigens were diluted to a concentration of 30 ng/mL in nasal swab material and then captured as described above.

## Supplementary Material

Supplementary File

## Data Availability

All study data are included in the article and/or *SI Appendix*.
