# Supplementary material for: Optofluidic multiplex detection of single SARS-CoV-2 and influenza A antigens using a novel bright fluorescent probe assay
Source: Proc Natl Acad Sci U S A. 2021 May 4;118(20):e2103480118. doi: 10.1073/pnas.2103480118 (PMC8158013; doi:10.1073/pnas.2103480118)
Supplement: Supplementary File [file pnas.2103480118.sapp.pdf]

PNAS
www.pnas.org

Supplementary Information for

Optofluidic multiplex detection of single SARS-CoV-2 and Influenza A antigens using a novel bright fluorescent probe assay

Alexandra Stambaugh, Joshua W. Parks, Matthew A. Stott, Gopikrishnan G. Meena, Aaron R. Hawkins, and Holger Schmidt

Alexandra Stambaugh
Email: amstamba@ucsc.edu

This PDF file includes:

Figures S1 to S3

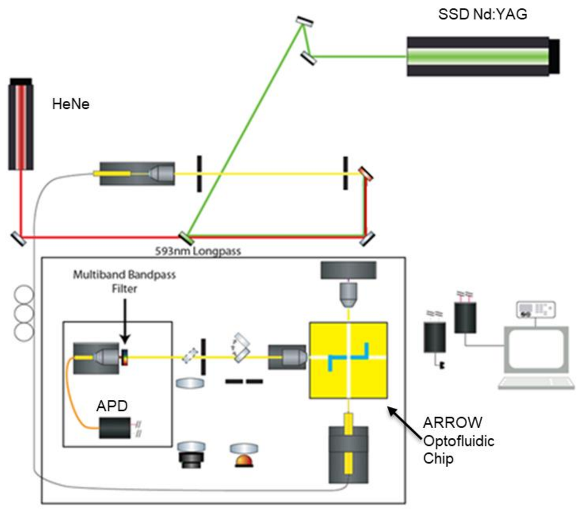

Fig. S1. Single Molecule detection setup. A 633 nm HeNe laser and a 556 nm SSD Nd:YAG laser are coupled into the same single mode fiber. The fiber is butt-coupled into the ARROW optofluidic chip single-mode excitation waveguide, which launches into a multimode excitation waveguide. Fluorescently tagged analytes flow through the ARROW channel (blue) and are orthogonally excited. They produce fluorescence signals, which are carried by the solid-core waveguide and coupled to a collection objective. The signal passes through a multi-bandpass filter before it is collected by an APD.

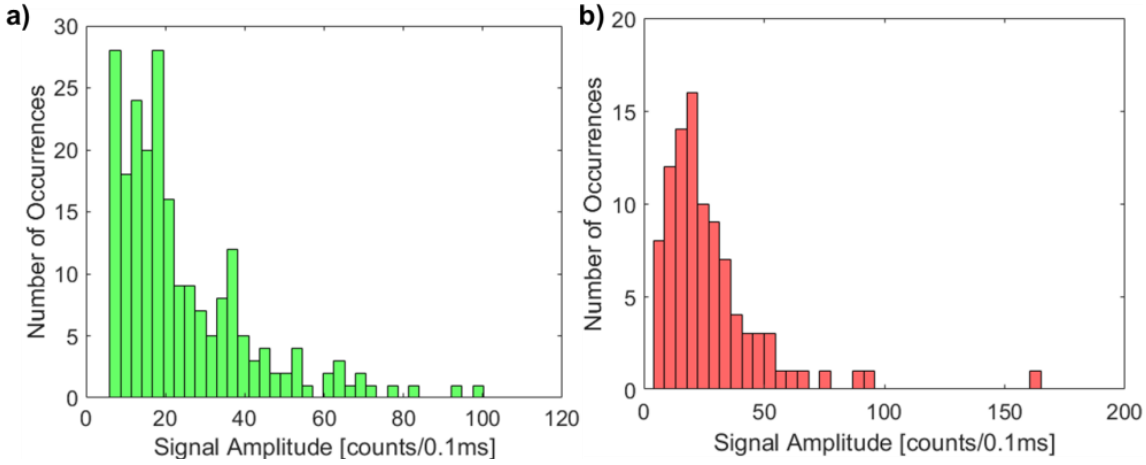

Fig. S2. Peak Amplitude Histograms of Single Probes a) Peak Amplitude Histogram of the 556 nm single color trace of Fig 4. (a) of the fluorescent probes in the main experiment, post capture in negative nasal swab material and post 45 second release experiment that have a mean signal amplitude of 24.2 counts/0.1ms and a standard deviation of 17.2 counts/0.1ms b) Peak Amplitude Histogram of the 633 nm single color trace of Fig 4. (a) of the fluorescent probes in the main experiment, post capture in negative nasal swab material and post 45 second release experiment that have a mean signal amplitude of 27.2 counts/0.1ms and a standard deviation of 22.2 counts/0.1ms.

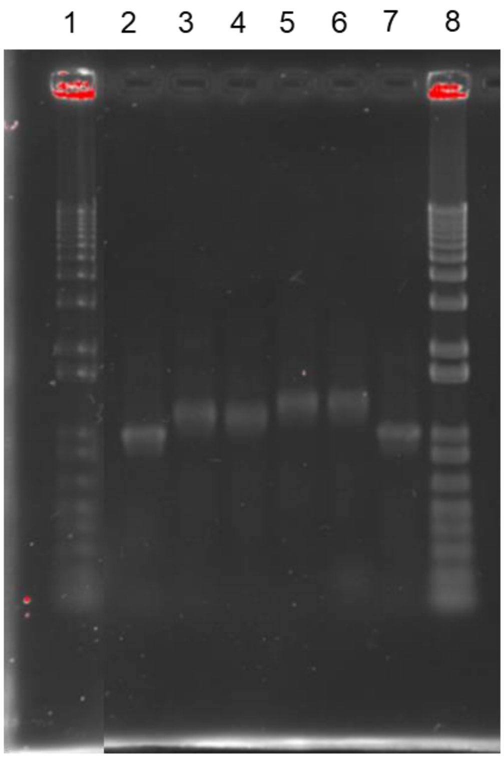

Fig. S3. 1% Agarose Gel Electrophoresis of Biotinylated PCR products. Lane 1: 1kB ladder, Lane 2: 0% Biotinylated-dUTP incorporation (control), Lane 3: 10% Biotinylated-dUTP incorporation, Lane 4: 25% Biotinylated-dUTP incorporation, Lane 5: 50% biotinylated-dUTP incorporation, Lane 6: 75% Biotinylated-dUTP incorporation, Lane 7: 0% Biotinylated-dUTP incorporation (control), Lane 8: 1kB ladder. The shift towards the top of the gel shows the level of biotin incoroporation: the shift in the 50% and 75% biotinylated-dUTP products shows a similar biotin incorporation into the probe backbone.
